# Effect of different mouthwash solutions on the surface morphology, nanohardness and flexural modulus of nickel-titanium orthodontic wire

**DOI:** 10.12669/pjms.40.9.9428

**Published:** 2024-10

**Authors:** Nozha Mahmoud Sawan, Afnan A Ben Gassem

**Affiliations:** 1 Nozha Mahmoud Sawan, BDS, M.Ed, MDS, Dip (ABO), Associate Professor, Department of Preventive Dental Sciences, College of Dentistry, Princess Nourah bint Abdulrahman University, Riyadh 11671, Saudi Arabia; 2 Afnan A Ben Gassem, BDS, M.Clin Dent, M.Ortho RCS, FHEA, Ph.D. Associate Professor, Department of Preventive Dentistry, College of Dentistry, Taibah University, Almadinah Amunawwara 42353, Saudi Arabia

**Keywords:** NiTi, arch wire, Mouthwash, Surface roughness, Nanohardness, Flexural modulus

## Abstract

**Objective:**

This laboratory study aimed to evaluate the effect of different mouthwash solutions on the surface and mechanical properties of NiTi arch wire.

**Methods:**

This experimental study was conducted at the Department of Preventive Dental Sciences, College of Dentistry, Princess Nourah bint Abdulrahman University, Saudi Arabia from September 2023 to November 2023. A 30 mm NiTi wires in length with 0.017 × 0.022” in dimensions were selected and equally divided into four groups: Control (G_0_) group wires were immersed in distilled water (DW); G_1_ wires were immersed in antiseptic mouthwash; G_2_ wires were immersed in fluoridated mouthwash; and G_3_ wires were immersed in therapeutic mouthwash. Surface morphology, nanohardness, and flexural modulus were evaluated at 24-hours, four weeks, and eight weeks’ time points. Data were statistically analyzed using a two-way analysis of variance (P<0.05).

**Results:**

The arch wires immersed in DW showed insignificant effects on surface roughness, nanohardness, and flexural modulus at different time points. However, all the experimental groups showed a significant effect of time and immersion solutions on the said properties (P<0.05). At the end of eight weeks, G_3_ showed the highest surface roughness (0.346 ± 0.032 µm) and the lowest nanohardness (1.350 ± 0.412 GPa) and flexural modulus (3.960 ± 0.140 MPa) compared to other study groups.

**Conclusions:**

The efficiency of tooth realignment could be influenced by the detrimental influence of fluoride and hydrogen peroxide mouthwash solutions on superelastic NiTi arch wires.

## INTRODUCTION

The available literature suggests that individuals with misaligned teeth face an elevated risk of periodontal issues due to the challenges associated with cleaning hard-to-reach areas.^1^ Tooth crowding further complicates the maintenance of proper oral hygiene.^2^ Moreover, compromised oral hygiene exacerbates staining, plaque formation, gum inflammation, enamel demineralization, and dental caries, particularly in proximity to orthodontic brackets. To meet treatment requirements and safeguard oral health, orthodontists frequently recommend the use of mouth rinses as a supportive measure for sustaining a favourable oral environment.^3^

The orthodontist prescribes several mouthwashes during orthodontic treatment, depending on the specific needs and circumstances of the patient. To reduce the risk of bacterial growth and gum inflammation, antiseptic mouthwashes containing chlorhexidine^4^ is used. To remineralize teeth, fluoridated mouthwash containing fluoride^5^ is advised. For teeth whitening, therapeutic mouthwashes containing hydrogen peroxide^6^ is prescribed and to control plaque formation, anti-plaque mouthwashes^4^ containing essential oils are usually recommended.

Fixed arch wires are typically made of nickel-titanium (NiTi), which is mainly composed of nickel and titanium with trace amounts of cobalt and chromium.^7^ The arch wires employed are designed such that to withstand corrosion within the oral environment due to a protective thin oxide film on their surface. Nevertheless, this safeguarding film is susceptible to both mechanical and chemical imbalances. The acidity of certain mouthwashes utilized during orthodontic treatment can potentially induce corrosion in the metallic elements of the arch wires, consequently modifying their physical, mechanical, and chemical attributes.^3^ The presence of acidic conditions and chloride ions can accelerate the degradation process of an oxide layer, ultimately causing corrosion of the wires within the oral cavity.^8^ This corrosion process results in surface irregularities of the wires, which weaken and can lead to the partial or complete dissolution of the wire, causing ion release in the oral cavity.^9^

Due to the change in the surface and mechanical properties of arch wires, friction, tooth movements, tissue compatibility, and aesthetics are compromised.^10^,^11^ It is believed that the deflection ability of ortho wires is also affected due to the corrosion process and ion release. Deflection of the wires is the ability to transmit forces to the dentoalveolar to promote controlled tooth movement.^12^ Additionally, wires with good deflection properties are more comfortable for patients. They exert gentle, consistent forces on the teeth, reducing discomfort and the potential for pain during treatment. Properly deflected wires can lead to more efficient treatment. They can facilitate quicker tooth movement^13^, potentially reducing the overall treatment duration.

Therefore, this laboratory study aimed to evaluate the effect of different mouthwashes on NiTi arch wires in terms of surface topography and morphological changes at different time points. Additionally, the effect of these mouthwashes was evaluated on the load/deflection test on the wires. It was hypothesized that the ortho wires would behave differently in different mouthwash solutions due to the compositional change of the mouthwash.

## METHODS

This experimental study was conducted at the Department of Preventive Dental Sciences, College of Dentistry, Princess Nourah bint Abdulrahman University, Saudi Arabia from September 2023 to November 2023. IRB exemption was obtained as this study fell under the category of a materials-based investigation. This experimental laboratory study used orthodontic NiTi wires with 0.017 × 0.022” in dimensions (Ortho Organizer, USA) that were sectioned into 30 mm in length from the straight portion of the arch wire. According to G*Power software for sample size calculation, a minimum of eight samples per group was required to achieve the statistical power for detecting a true effect. Three mouthwash solutions with different formulations and compositions were selected (Table-I). A total of thirty-two arch wires were broadly divided into four study groups as follows:

**Table-I:** Composition of mouthwash products used and their primary use.

Mouthwash	Ingredients	Primary use
Periogard^®^	Chlorhexidine Gluconate (0.12%), water, glycerin, ethanol, polysorbate 20, mint flavour aromatic composition, sodium saccharin	Antiseptic and germicidal
LISTERINE^®^ Total Care	Water, ethanol, menthol, eucalyptol, thymol, methyl salicylate, benzoic acid, poloxamer 407, fluoride, zinc chloride, and flavour	Enamel mineralization
Colgate Optic^®^ White Whitening	Water, Glycerin, Propylene Glycol, Sorbitol, Hydrogen Peroxide, Polysorbate 20, Sodium Acrylates/Methacryloylethyl Phosphate Copolymer, Phosphoric Acid, Citric Acid, Flavor, PVM/MA Copolymer, Sodium Saccharin	Therapeutic mouthwash for teeth whitening

- Group-1 (G_1_) was designated as the control group. The sample wires were continuously immersed in artificial saliva and incubated at 37ºC with 100% humidity.
- Group-2 (G_2_) sample wires were subjected to a treatment regimen that included immersion in artificial saliva, followed by two daily sessions of 90 s immersions in antiseptic mouthwash (Periogard^®^). Subsequently, the wires were re-immersed in artificial saliva and stored in an incubator at 37°C with 100% humidity.
- Group-3 (G_3_) sample wires were immersed in distilled water but were immersed in the fluoridated mouthwash (LISTERINE^®^ Total Care) twice a day for 90 s; subsequently the wires were returned into artificial saliva and incubated at 37ºC with 100% humidity.
- Group-4 (G_4_) sample wires were immersed in artificial saliva but were immersed in the therapeutic mouthwash (Colgate Optic^®^ White Whitening) twice a day for 90 s; subsequently, the wires were returned into distilled water and incubated at 37ºC with 100% humidity. Each group was further divided into three subgroups based on the duration of observation, specifically at baseline (24-hours), four and eight weeks, with each subgroup containing eight arch wires.

### Surface roughness test:

The surface topography was performed using a non-contact surface profiler, i.e., Contour GT-K 3D Optical Microscope (Bruker®, Germany) with interferometry. Samples were measured by Vertical Scan Interferometry using a 5x Michelson magnification lens with a field of view of 1 mm x 1 mm, a Gaussian Regression Filter to suppress noise, a scan speed of 1x, and a thresholding of four.

The wires were placed on the stage and manually adjusted for optimal position. The microscope used a Vision 64 (Bruker®) software which controlled the instrument settings, data analyses, and graphical output. The measurement was performed using vertical scanning interferometry which uses a broadband (normally white) light source which is effective for measuring objects with rough surfaces, as well as those with adjacent pixel-height differences greater than 135 nm. Each sample was scanned at three intervals and averaged accordingly to determine the roughness (Ra) value.

### Nanohardness test:

To gauge the effect of immersing mouthwash solutions on arch wires, a set of 8 wires 30 mm long each was evaluated for nanohardness test using a nanomechanical tester (UMT1, Bruker, Santa Barbara, CA, USA) with a Berkovich diamond indenter tip with 100 nm radius. The tests were conducted at a room temperature of 23°C, with loading and unloading rates of 2.0 mN/s and a dwell time of 10 Seconds. The maximal load was set to 20.0 mN. Three readings on each sample were measured and the mean value of nanohardness for each sample was computed.

### Flexural modulus test:

A universal testing machine (Instron 5967, Instron, Canton, MA, USA) was used to perform the 3-point flexural modulus test. Each sample wire was mounted on a custom-made epoxy mould that supported two metal brackets secured with rubber ties with an inter-bracket distance of 14 mm. A downward speed rate of 10 mm/min was applied on the 0.022 side of the wire to cause deflection for a distance of 2mm. Maximum load to cause displacement was registered in Newton.

### Scanning electron microscope (SEM) evaluation:

Following a cleaning process involving a 12 minutes ultrasonic bath and a subsequent five min air blast, the wires were introduced into the stage of scanning electron microscope (SEM, JEOL JSM-6610LV, Japan) for image processing. The instrumental parameters employed included an electron beam energy of 15kV with a working distance of 10mm, and a magnification of 1000x was applied.

### Statistical analysis:

The data collected underwent tabulation and analysis through SPSS Software version 28.0. Dependent variables were described using mean and standard deviation. To ascertain the effect of three distinct mouthwash solutions at three different time points (*i.e*., 24-hours, four weeks, and eight weeks) on arch wires, a two-way analysis of variance (ANOVA) was deployed followed by Bonferroni’s *post hoc* multiple comparison tests. Throughout all statistical examinations, a probability value of 0.05 or less was considered statistical significance.

## RESULTS

The surface roughness values of the study groups (in µm) are reported in Table-II. The baseline (i.e., 24-hours) readings of the study groups showed approximate surface roughness values. Notably, we witnessed a negligible and gradual increase in surface roughness for the control group (G_0_) spanning from the 24-hours’ time point to the eight weeks’ time point. In contrast, the experimental groups displayed a statistically significant increase in surface roughness either from the 24-hours’ time point to the four weeks’ time point or from the four weeks’ time point to the eight weeks’ time point (p < 0.05). At the end of eight weeks, the maximum surface roughness was observed in the G_3_ group (0.346 ± 0.032 µm) while G_0_ showed the least (0.181 ± 0.009 µm).

**Table-II:** Mean surface roughness (in µm) of the study groups based on mouthwash and immersing conditions used.

Group (n=8)	Surface roughness (µm)		

	24-hours	four weeks	eight weeks
G_0_	0.174 ± 0.006	0.176 ± 0.005	0.181 ± 0.009
G_1_	0.173 ± 0.005_a_	0.227 ± 0.051	0.277 ± 0.053_a_
G_2_	0.171 ± 0.010_b,c_	0.278 ± 0.024_b,d_	0.335 ± 0.367_c,d_
G_3_	0.165 ± 0.013_e,f_	0.310 ± 0.011_e,g_	0.346 ± 0.032_f,g_

***Key:*** The subscript notations show significant differences within the mouthwash group.

The mean and standard deviation nanohardness values of the study groups (in GPa) are reported in Table-III. The baseline (i.e., 24 hours) readings of the study groups showed approximate nanohardness values. Over time, a gradual insignificant decline in nanohardness was witnessed within the control group, i.e., G_0_ (p >0.05). Conversely, the experimental groups presented significant decrease in nanohardness values either from the 24 hours’ time point to the four weeks’ time point or from the four weeks’ time point to the eight weeks’ time point (p < 0.05). At the end of eight weeks, the lowest nanohardness was detected in the G_3_ group (1.350 ± 0.412 GPa) compared to G_0_, which exhibited the highest (2.179 ± 0.362 GPa).

**Table-III:** Mean nanohardness (in GPa) of the study groups based on mouthwash and immersing conditions used.

Group (n=8)	Nanohardness (GPa)		

	24-hours	four weeks	eight weeks
G_0_	2.581 ± 0.331	2.384 ± 0.314	2.179 ± 0.362
G_1_	2.867 ± 0.401_a_	2.275 ± 0.651	2.093 ± 0.419_a_
G_2_	2.703 ± 0.385_b_	2.274 ± 0.514	1.774 ± 0.211_b_
G_3_	2.748 ± 0.318_c,d_	1.806 ± 0.259_c,e_	1.350 ± 0.412_d,e_

***Key:*** See Table-II.

Flexural modulus in Table-IV represents the values of the study groups (in MPa). The baseline (i.e., 24 hours) readings of the study groups showed estimated flexural modulus when the wires were deflected to 2 mm. Notably, a negligible change in flexural modulus was observed in the control group (G_0_) at different time points (p >0.05). However, the flexural modulus gradually decreased from 24 hours to four weeks and from four weeks to eight weeks. In contrast, the experimental groups displayed a significant decrease in flexural modulus values at each time point (p < 0.05). At the end of eight weeks, the highest reduction was observed in the G_3_ group (3.960 ± 0.140 MPa) while the control group exhibited the least (4.646 ± 0.325 MPa). SEM pictograms of the study groups over time are displayed in Fig-3..We observed a smooth surface without pitting and irregularities amongst the baseline group of the study groups (Fig.3A-3C). However, due to surface treatment and immersion media, surface microstructural changes amongst the study groups were observed at both four weeks and eight weeks’ time points. The qualitative surface roughness increases and the presence of pits and porosities was observed amongst G_1_, G_2_, and G_3_ (Fig.3D-3F). However, further increases in pitting and irregularities were observed at an eight weeks’ time point amongst G_2_ (Fig.3H) and G_3_ (Fig.3I) compared to G_0_ (Fig.3G).

**Table-IV:** Mean flexural modulus (in MPa) of the study groups based on mouthwash and immersing conditions used.

Group (n=8)	Flexural modulus (MPa)		

	24-hours	four weeks	eight weeks
G_0_	4.810 ± 0.184	4.769 ± 0.202	4.646 ± 0.325
G_1_	4.810 ± 0.212_a_	4.577 ± 0.193_b_	4.190 ± 0.194_a,b_
G_2_	4.707 ± 0.187_c,d_	4.249 ± 0.074_c,e_	3.986 ± 0.116_d,e_
G_3_	4.859 ± 0.263_f,g_	4.231 ± 0.177_f,h_	3.960 ± 0.140_g,h_

***Key:*** See Table-II.

**Fig. 1A & 1B:**
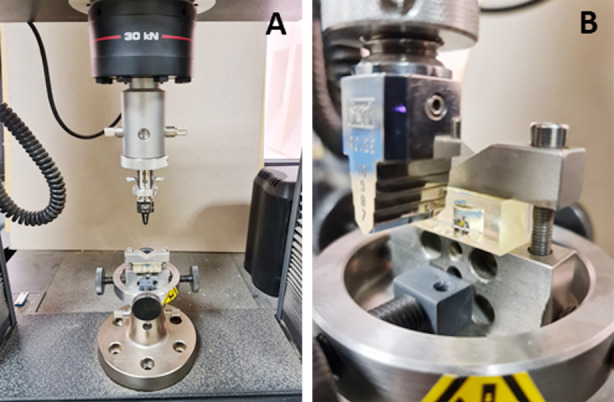
(A) Universal testing machine for load/deflection test; (B) Deflection test for arch wire with 0.017 × 0.022” in dimensions.

**Fig.2:**
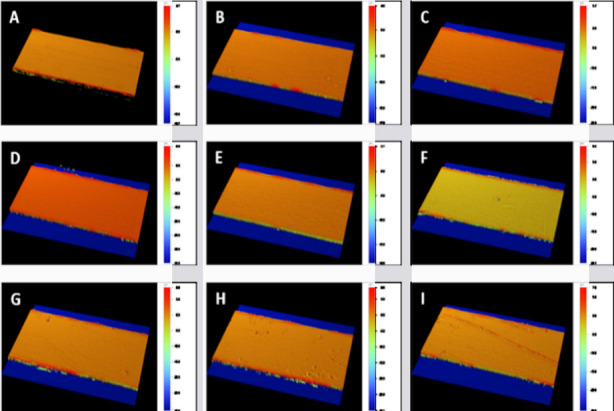
The surface profiles of orthodontic arch wires in different mouthwash solutions over time: (A-C) profilograms at baseline of G_0_, G_1,_ and G_3_ groups, respectively; (D-C) profilograms at four weeks of G_0_, G_1_ and G_3_ groups, respectively; and (G-I) profilograms at eight weeks of G_1_, G_2_ and G_3_ groups, respectively.

**Fig. 3:**
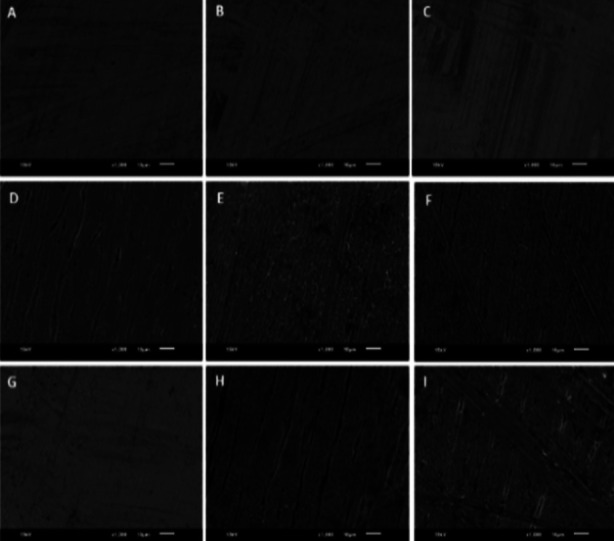
Surface microstructure of orthodontic arch wires in different mouthwash solutions over time: (A-C) microstructure at baseline of G_0_, G_1,_ and G_2_ groups, respectively; (D-C) microstructure at four weeks of G_1_, G_2_ and G_3_ groups, respectively; and (G-I) microstructure at eight weeks of G_0_, G_2_ and G_3_ groups, respectively.

## DISCUSSION

The results of this laboratory study indicated that arch wires exhibited varying responses when subjected to different mouthwash solutions, resulting in changes in surface roughness, nanohardness, and flexural modulus. This effect was particularly pronounced following extended exposures of either four weeks or eight weeks. Consequently, the data provides backing for the hypothesis that modifications in mouthwash composition can impact the behavior of these wires.

The surface roughness is a vital parameter for evaluating the arch wires. The higher roughness of arch wires may lead to plaque accumulation and frictional resistance.^14^ This friction generates increased impediment to the movement between the wires and brackets.^2^

Our data indicate that the surface roughness of the arch wires is influenced by the mouthwash used. Throughout the experiment, an insignificant increase in surface roughness of the control group may be attributed to distilled water possessing a pH of 7.5, confirming its neutrality.^15^ An insignificant increase in surface roughness could be due to the natural wear and tear of wires. However, a significant increase in surface roughness using antiseptic mouthwash may suggest that chlorhexidine, especially in acidic mouthwash solutions, may promote the dissolution of the nickel and the chromium ions of the NiTi,^7^ leading to surface pitting and roughening.^15^ Our finding is in accordance with the previous study^3^, however it contradicts with the findings of another previous study that advocated non-harmful effect of chlorhexidine-containing mouthwash on the NiTi wires.^16^

Additionally, the presence of fluoride content in mouthwash may induce the destruction of the titanium oxide layer of NiTi, resulting in the formation of titanium fluoride, titanium oxide fluoride, or sodium titanium fluoride on the surface.^17^ When the concentration of sodium fluoride ions surpasses 0.1 wt.%, it compromises the protective function of the titanium oxide layer that develops on the titanium alloy.^15^ Therapeutic mouthwash contains active ingredients such as chlorhexidine, fluoride, cetylpyridinium chloride, thymol and hydrogen peroxide. These active ingredients may cause passivating effect on wires.^18^ The findings are in line with the previous studies by Farrag et al.^16^ and Wajahat et al.^2^ who observed that sodium fluoride can alter surface properties of NiTi wires.

Hardness is an important physico-mechanical property. However, through nanohardness evaluation, the unit area of the indented surface at the nanoscale can be measured.^19^ The obtained data suggest deleterious effects of the active ingredients of the mouthwash solutions. The insignificant effect of artificial saliva was observed on the nanohardness of the study groups. A significant decrease in nanohardness using antiseptic, fluoridated, and therapeutic mouthwashes may suggest that all these mouthwashes with a low pH solution are acidic. The process of dissolution of metal ions takes place in an acidic environment and weakens the surface,^8^ making it softer and reducing the hardness.^20^

Additionally, the active ingredients of these mouthwashes such as chlorhexidine in antiseptic, fluoride in fluoridated, and hydrogen peroxide in therapeutic mouthwashes act as oxidizing agents, leading to corrosion of the metal surface.^3^ Similarly, fluoride ions have the potential to reach metallic surfaces, forming metal fluoride compounds that contribute to the loss of metal ions, corrosion, and subsequent reduction in hardness.^21^ While hydrogen peroxide causes oxidative effects on metal surfaces. It may facilitate the formation of metal oxides and promote corrosion.^22^ The current work used nanohardness parameter, which has not been previously investigated. Therefore, direct comparisons with previous studies are difficult due to the lack of comparable data.

By using the three-point flexural test, the stiffness of arch wires can be gauged. For a guided tooth movement, the stiffness of the wire plays a pivotal role. However, we observed that all the mouthwashes reduced the flexural modulus of the arch wires. The reasons for reduced flexural modulus are similar to those related to surface and nanomechanical changes due to ion release and surface degradation. These reasons have already been discussed earlier. A previous study by Mane et al. advocated that the wire exposure to fluoride can form a substance referred to as a “titanium hybrid.” This suggests that fluoride may interact with the titanium component of the NiTi wire.^23^ Another study by Pastor et al. has endorsed the perilous release of nickel ions. This release of nickel ions impedes super elasticity and transforms the wire into a non-functional component incapable of exerting corrective stress.^24^ Our research supports the findings of earlier investigations.^23^,^24^

The qualitative data pertaining to SEM evaluation present an intact and clean surface of the control group at all-time points (Fig. 3A & 3G). While the wires that were subjected to antiseptic and fluoridated mouthwash immersion showed white patches with globular, pitted, and elongated flaws.^18^ These pitting and globular flaws arise from the wire’s wrought surface.^18^ Exposure of wires to H_2_O_2_ causes penetration of hydrogen into interstitial spaces, grain boundaries, and cracks, where it reacts with lattice atoms to create titanium hydride. This process leads to the formation of a body-centred tetragonal structure, which is believed to have an impact on the material’s mechanical properties.^3^,^25^ The SEM data corroborate well with the mechanical analysis of the wire.

The orthodontists ought to proceed with caution since various formulations of mouthwashes can significantly change the mechanical attributes and surface morphology of NiTi wires, thus compromising the effectiveness and results of orthodontic treatment. This research advances our understanding of the mechanisms behind the detrimental effects of various mouthwash formulations, especially those that contain hydrogen peroxide and fluoride, have on the flexural modulus, nanohardness, and surface roughness of NiTi orthodontic wires.

Laboratory experiments are often performed in controlled and simplified conditions that may not replicate the complex and dynamic oral environment. Considerable variations were observed across the available studies in terms of mouthwash solution, active agent concentration, solution pH levels, immersion durations, the mechanical properties assessed, and the methodologies employed. These disparities hindered the possibility of making direct comparisons among the results. In the future, studies related to the investigation of specific ingredients in mouthwash formulations that contribute to changes in arch wire properties would be beneficial.

## CONCLUSION

Mouthwashes may be prescribed cautiously during orthodontic treatment. All mouthwashes altered the mechanical properties as well as the surface morphology of 0.017 × 0.022” wires, potentially influencing the mechanical characteristics of the wires throughout orthodontic treatment.
